# Supplementary material for: Smoking Habit and Respiratory Function Predict Patients’ Outcome after Surgery for Lung Cancer, Irrespective of Histotype and Disease Stage
Source: J Clin Med. 2023 Feb 16;12(4):1561. doi: 10.3390/jcm12041561 (PMC9967492; doi:10.3390/jcm12041561)
Supplement: Supplementary file 1 [file jcm-12-01561-s001.zip › jcm-2216983-supplementary.pdf]

Surgical Techniques.

The typical segmentectomy was performed by removing one or more lung parenchymal segments with the corresponding broncho-vascular and lymphatic supply. The atypical resection (which does not involve anatomical dissection of the hilum) was performed by using the endostapler directly on the parenchyma by making a wedge cut. The endostapler was then applied while maintaining a parenchymal resection margin on the adjacent segment of the lung (extended segmentectomy) ensuring a resection margin equivalent to the diameter of the tumor. Individual isolation, ligation and division of the corresponding bronchovascular contributions to the lobe was also performed for the lobectomy, similar to the technique used for typical segmentectomy. The distribution of resected segments and lobes is provided in supplementary Table 1. For all the techniques, systematic ilo-mediastinal lymph node dissection was performed at the end of the lung resection. Chest drainage management and discharge planning were individualized based on the patient's clinical characteristics and the surgeon's judgment. In case of prolonged air leaks, patients were discharged after placing a Heimlich valve and ensuring the stability of lung expansion. Discharge criteria were not influenced by the type of surgery performed.

Supplementary Table S1. Distribution of the resected lobes and segments. RUP= right upper lobe, LUL= left upper lobe, RML= right medial lobe, RLL= right lower lobe, LLL= left lower lobe.

Resection	Lobectomy	%	Typical SG	%	Atypical SG	%
RLL	19	18,45%	0	0,00%	0	0,00%
LLL	24	23,30%	0	0,00%	0	0,00%
RML	10	9,71%	0	0,00%	0	0,00%
RUL	28	27,18%	0	0,00%	0	0,00%
LUL	22	21,36%	0	0,00%	0	0,00%
S1-2-3, apico-posterior, anterior LUL	0	0,00%	0	0,00%	1	4,17%
S1-2, apical, posterior RUL	0	0,00%	1	3,85%	0	0,00%
S1-2, apico-posterior LUL	0	0,00%	3	11,54%	1	4,17%
S1-2, apico-posterior RUL	0	0,00%	1	3,85%	1	4,17%
S1, apical RUL	0	0,00%	3	11,54%	3	12,50%
S1, apical LUL	0	0,00%	2	7,69%	5	20,83%
S10, posterior RLL	0	0,00%	1	3,85%	1	4,17%
S2-3, posterior LUL	0	0,00%	0	0,00%	1	4,17%
S2, posterior RUL	0	0,00%	4	15,38%	4	15,38%
S2, posterior LUL	0	0,00%	3	11,54%	1	4,17%
S3, anteriori RUL	0	0,00%	1	3,85%	2	4,17%
S3, anterior LUL	0	0,00%	4	11,54%	1	4,17%
S5, lingular LUL	0	0,00%	1	3,85%	0	0,00%
S5, medial RML	0	0,00%	1	3,85%	0	0,00%
S6, apical RIL	0	0,00%	0	0,00%	2	8,33%
S6, apical LIL	0	0,00%	1	3,85%	1	4,17%
	**103**	**100,0%**	**26**	**100,00%**	**24**	**100,00%**

Supplementary Table S2. Preoperative functional data and data regarding resected lymphnodes Pres= pre-surgey ; pps= predicted post operative.

	Lobectomy Average (SD)	Typical SG Average (SD)	Atypical SG Average (SD)
FEV1 pres %	88,33 (19,5)	87.778 (18,66)	78,9 (22,9)
DLCO preop%	78,71 (16,42)	77.529 (18,12)	74,8 (19,27)
ppsFEV1	1,84 (0,63)	1.750 (0,58)	1,64 (0,5)
ppoDLCO %	62,39 (13,86)	60.588 (13,32)	59,46 (15,8)
Total number of resected lymphnodes	8,19 (3,016)	5,15 (2,55)	3,91 (2,37)
Total number of resected lymphatic stations	2,72 (1)	2,61 (0,94)	1,75 (0,94)

Supplementary Table S3. Detailed data regarding surgical margins.

	Lobectomy	%	Typical SG	%	Atypical SG	%
Invasion of the surgical margin	0	0%	3	11,5%	3	12,5%
Surgical margin <1 cm	22	21,35%	19	73%	14	58,3%
1< surgical margin < 2 cm	18	17,49%	3	11,5%	7	29,2%
Surgical margin >2 cm	63	61,16%	1	4%	0	0%
Total	**103**	**100%**	**26**	**100%**	**24**	**100%**

Supplementary Table S4. Data regarding tumor relapse after the three different surgical approaches.

	Lobectomy	%	Typical SG	%	Atypical SG	%
Total relapses	16		5		7	
Relapse <1 year	3	18,75%	3	60%	4	57,15%
1< Relapse <2 years	4	25%	2	40%	0	0%
2< Relapse <3 years	4	25%	0	0%	2	28,57%
3< Relapse <4 years	1	6,25%	0	0%	1	14,28%
4< Relapse <5 years	4	25%	0	0%	0	0%
		**100%**		**100%**		**100%**

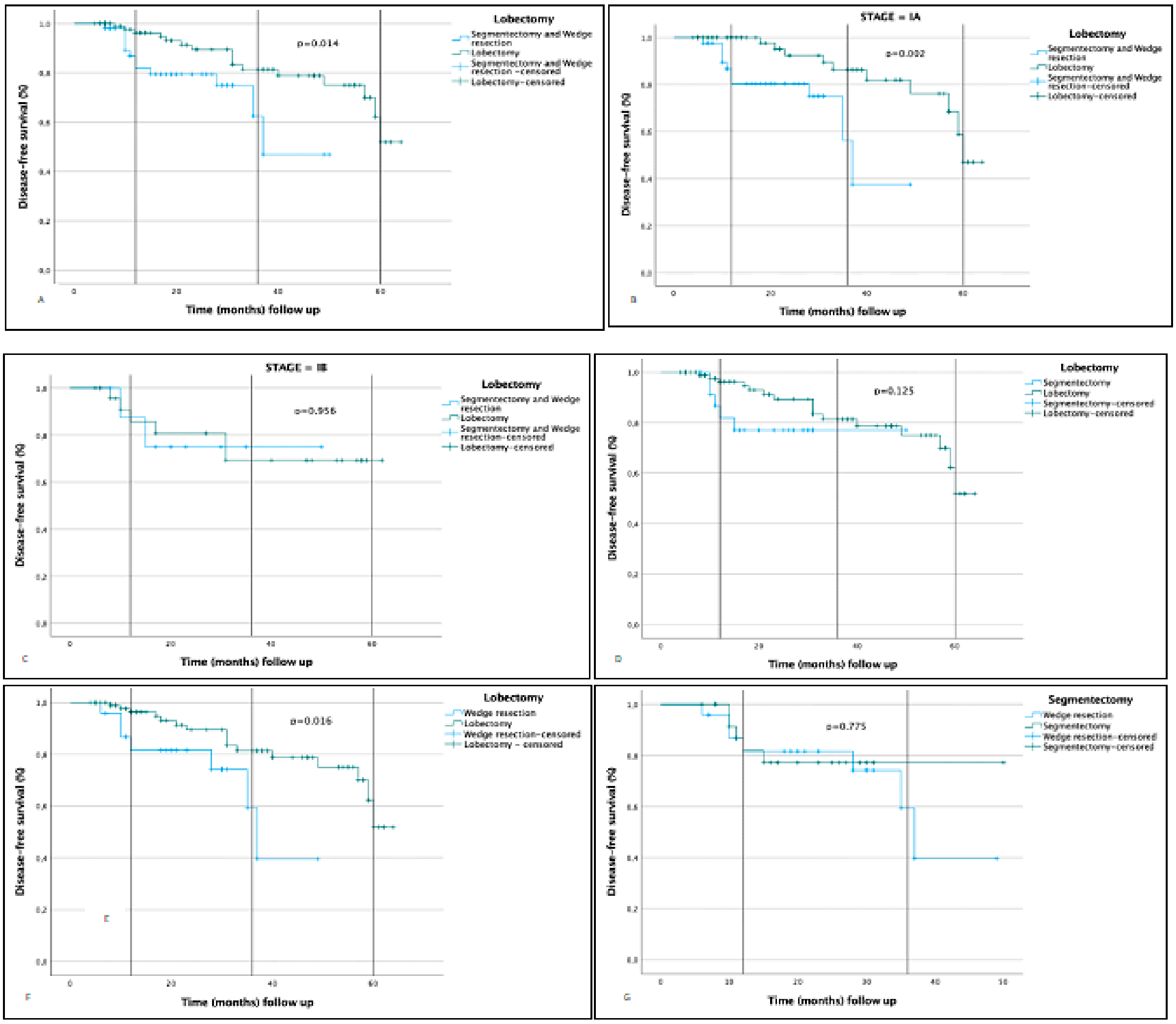

Supplementary Figure S1. Cumulative DFS % compared between lobectomy and the two types of segmentectomy in stage IA (**A**) and IB diseases (**B**), respectively.
